# WW domain-binding protein 2 acts as an oncogene by modulating the activity of the glycolytic enzyme ENO1 in glioma

**DOI:** 10.1038/s41419-018-0376-5

**Published:** 2018-03-01

**Authors:** Shuai Chen, Ya Zhang, Han Wang, Yu-Ying Zeng, Zhi Li, Ming-Li Li, Fang-Fang Li, Jun You, Zhi-Ming Zhang, Chi-Meng Tzeng

**Affiliations:** 1 Translational Medicine Research Center (TMRC), School of Pharmaceutical Science, Xiamen University, Xiamen, Fujian 361005 P.R. China; 2 Department of Breast Surgery, The First Affiliated Hospital of Xiamen University, Xiamen, Fujian 361005 P.R. China; 3 INNOVA Cell Theranostics/Clinics and TRANSLA Health Group, Yangzhou, Jiangsu P.R. China; 4 Key Laboratory for Cancer T-Cell Therapeutics and Clinical Translation (CTCTCT), Xiamen, Fujian, 361005 P.R. China; 5 College of Pharmaceutical Sciences, Nanjing Tech University, Nanjing, 211816 China; 6 Jiansu Provincial Institute of Translation Medicine and Women-Child Health Care Hospital Affiliated to Nanjing Medical University, Nanjing, 210029 China; 7 Teaching Hospital of Fujian Medical University, Fuzhou, Fujian 350004 P.R. China

## Abstract

WW domain-binding protein 2 (*WBP2*) has been demonstrated as oncogenic in breast cancer. Many studies have revealed the *WBP2* gene as a high-risk gene for leukoariaosis and cerebral white matter lesions is important in the pathologic stage of glioma development. This study aimed to illustrate the underlying mechanism by which WBP2 regulates the process of glioma development. The expression pattern of WBP2 in several tumor cells was determined, clarifying the carcinogenic action of WBP2 in glioma cells. Overexpression of WBP2 in glioma cells promoted cell proliferation and migration, and the number of S-phase cells, whereas the depletion of WBP2 by RNAi-mediated knockdown restrained cell growth and cell cycle progression. Upregulation of WBP2 significantly enhanced the tumorigenic ability of U251 cells *in vivo*. MS/GST pulldown assay identified α-enolase (*ENO1*) and Homer protein homolog 3 (*Homer3*) as novel potent interaction partners of WBP2. Knockdown of ENO1 or Homer3 allowed cell growth and migration to return to normal levels. Furthermore, *in vitro* and *in vivo* experiments indicated that the oncogenic role of WBP2 in glioma was through modulating ENO1 and glycolysis activity via the ENO1-PI3K/Akt signaling pathway. Collectively, these results reveal that WBP2 plays a vital role in the occurrence and development of glioma, indicating a target gene for glioblastoma treatment.

## Introduction

Malignant glioma is characterized as a highly aggressive cancer and the most dangerous type of primary brain tumor occurring in the central nervous system^1^. Surgical resection of malignant glioma is rarely successful because the tumor nodes infiltrate surrounding normal tissue^2^. In recent years, progress has been made in improving diagnostic methods and therapeutic strategies for glioma, but there is still no successful treatment for highly malignant gliomas^3^.

Glucose metabolism disorder of cells is a typical feature in tumorigenesis^4^. In common with other cancers, glioblastomas uniquely produce energy through aerobic glycolysis, an observation known as the Warburg effect^5^. Recent studies have suggested that compensatory mechanisms, such as the absorption of glucose and glycolytic activity, thrive in malignant glioma cells^6^. The enolase ENO1 (α-enolase) is a glycolytic enzyme responsible for the conversion of 2-phosphoglycerate to phosphoenolpyruvate and functions in aerobic glycolysis, contributing to the Warburg effect in tumor cells^7^. ENO1 expression is detected in most tissues and its overexpression is associated with multiple tumors, including glioma, neuroblastoma, and other types of cancers^6–9^. Previous studies have indicated that α-enolase, as a potential cancer prognostic marker, enhances cell growth, migration, and invasion progression by activating the PI3K/Akt pathway in glioma cells^6^. Moreover, ENO1 as a plasminogen receptor on the tumor cell surface could induce extracellular matrix degradation, tumorigenesis, and cancer invasion during pathologic conditions^10^. Considering these factors, ENO1 may be a potent therapeutic target for treating malignant glioma patients.

WW domain-binding protein 2 (*WBP2*) has three PPXY motifs at C terminal that have been identified as being involved in protein–protein interactions through binding with WW-domain-containing molecules^11^. As a tyrosine kinase substrate, tyrosine phosphorylation of WBP2 at Try192 and Try231 stimulated by epidermal growth factor (EGF) can cause disturbances of cell proliferation regulation and induce tumorigenesis in breast cancer cells^12^. WBP2 operates by regulating the expression of target genes via hormone‐dependent interaction^13^. It is confirmed that phosphorylated WBP2 can enter the nucleus and enhance the transactivation functions of the progesterone receptor and estrogen receptor (ER)^14^. WBP2 is required for normal glutamatergic synapses in the cochlea and is involved in the molecular pathway linking hearing impairment to hormonal signaling^15^. Genome-wide association studies of cerebral white matter lesions indicate that *WBP2* gene is high risk for leukoaraiosis, suggesting that WBP2 might be a key regulator of nervous system inflammation^16^. The relationship between inflammation and cancer is established and studies show that WBP2 expression can enhance the proliferation and metastatic ability of breast cancer cells^17,18^; however, to our knowledge, the expression and function of WBP2 in glioma has not been reported.

We evaluated the expression of ENO1 in several cancer cell lines and found that ENO1 and Homer3 were potent partners of WBP2 in U251 cells. ENO1 is a hub protein in the Embden–Meyerhof–Parnas (EMP) pathway providing energy for glioma tumor cells. Homer3, a member of the Homer family of scaffold proteins, can regulate transcription and plays a critical role in the differentiation and development of the nervous system^19,20^. However, the cross-talk between ENO1, Homer3, and WBP2 remains poorly understood in the progression of glioma. The results presented here will reveal the relationship between these proteins and their role in the oncogenesis of glioma.

## Results

### WBP2 is highly expressed in human glioma

**Fig. 1:**
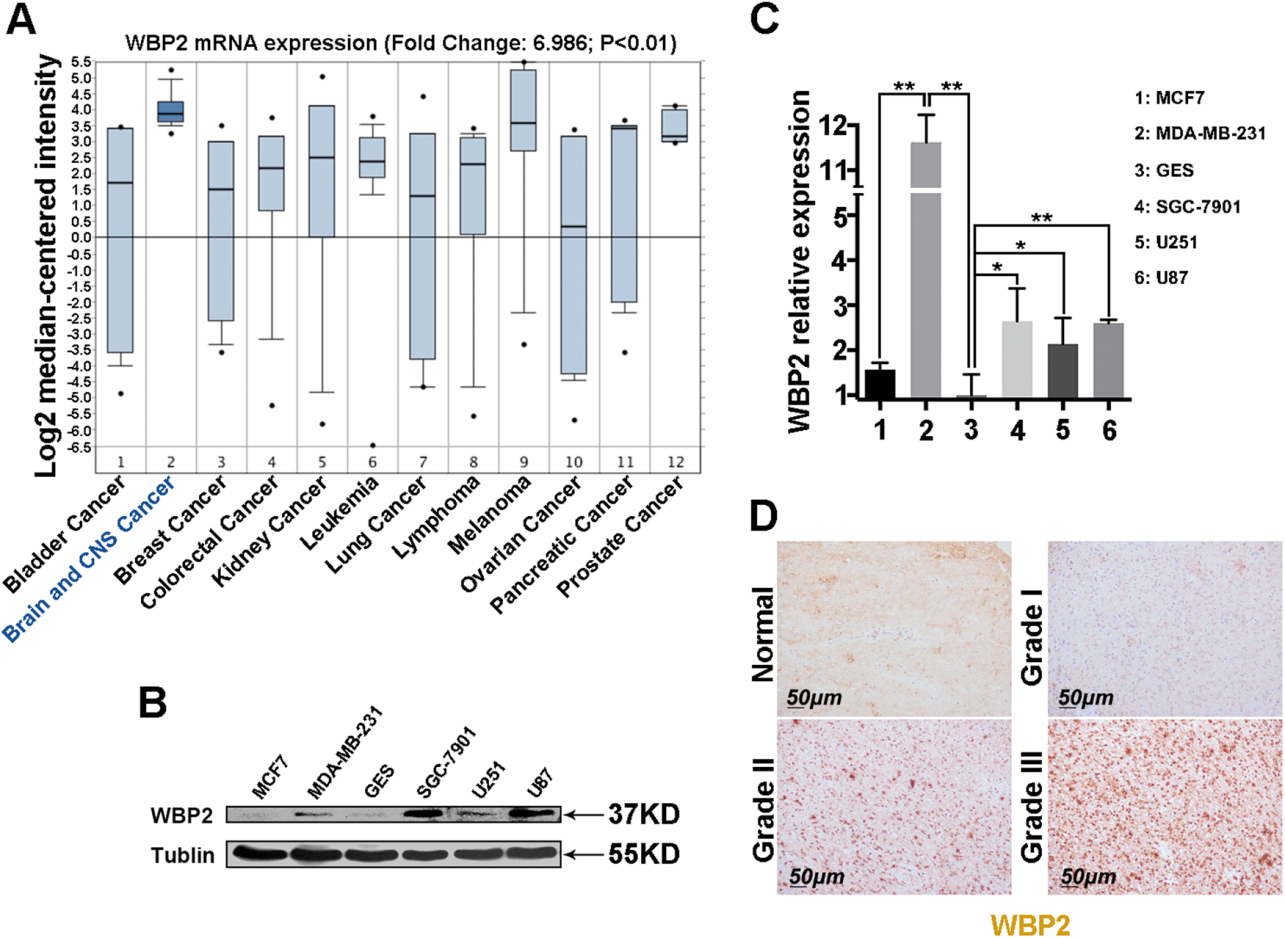
Clinical and cellular significances of WBP2 in human glioma. **a** Oncomine data mining analysis of *WBP2* mRNA level in Ramaswamy Multi-cancer data sets among 12 types of cancer. **b** and **c** The differential protein and transcriptional expression of WBP2 in breast cancer (MCF7 and MDA-MB-231 cells), gastric cancer (GES and SGC-7901 cells), and glioma cell lines (U251 and U87). **d** Immunohistochemical staining of WBP2 in glioma tissue microarray. Normal, Normal brain tissue. Grade I, II, and III indicate pathologic grades of glioma samples. Scale bar, 50 μm. *P < 0.05, **P < 0.01

Previous studies have shown that WBP2 acts as an oncogene in breast cancer^21^, but there is not yet any published evidence of its carcinogenesis in the nervous system. To determine the clinical significances of WBP2 in patients with brain and CNS cancer, we performed data mining and analyzed *WBP2* mRNA expression pattern from the publicly available Oncomine database. Based on the Ramaswamy Multi-Cancer Statistics (20 of 169 samples was brain and CNS cancer cases), WBP2 was observably upregulated in brain and CNS cancer in comparison with other types of cancer (Fig. 1a). These results raise the possibility that WBP2 have functional correlation with human brain cancer. Then, we also detected the expression of WBP2 in several different tumor cell lines including breast cancer (MDA-MB-231 and MCF7), gastric cancer (SGC7901), glioma cells (U87 and U251), and in a strain of normal cells, gastric epithelial cells (GES-1), and found that WBP2 protein and mRNA levels were upregulated in the highly invasive tumor cells MDA-MB-231, SGC-7901, U87, and U251, in comparison with the less invasive cell lines MCF7 and normal cell line GES-1 (Fig. 1b-c). When considering the role of WBP2 in cerebral white matter lesions, we focused on the relationship between WBP2 and glioma. Because of its expression pattern in glioma cell lines, we suspected WBP2 may act as a carcinogenic gene in glioma. To verify the expression pattern of WBP2 in glioma, we performed immunohistochemical (IHC) staining with WBP2 antibody to evaluate WBP2 protein levels, using tissue microarray. The characteristics of the microarray samples are presented in Table 1. Samples from three normal human brains and 72 human brains with glioma were used. The immunohistochemistry results (Fig. 1d) showed that WBP2 was highly expressed in 68% of the glioma samples. Moreover, 41 samples from patients defined as having grade III glioma exhibited high expression of WBP2. Glioma classification and staging is currently determined on the basis of the presence or absence of IDH1/2 mutations, which are associated with the metabolic reprogramming of glioma resulting in a different metabolism compared to patients with wild-type IDH. Based on this, we performed IHC staining with primary antibody against IDH1/2 mutant and found 20 samples with IDH1/2 mutation in the same tissue microarray. The IDH1/2 mutant positive staining were showed in the supplementary information (Fig. S1). To validate the expression pattern of WBP2 in wild-type glioma, we excluded the 20 specimens with IDH1/2 mutation and found that WBP2 was highly expressed in 78.2% of wild-type glioma samples (43/55), in which 36 samples defined as grade III glioma presented high WBP2 expression (Table 1). These data suggest that WBP2 is upregulated in glioma cells and human glioma tissues without IDH1/2 mutation and may be an underlying carcinogenic factor in neuroglioma.

**Table 1:** Correlation between WBP2 expression and clinicopathological characteristics of Glioma patients

Characteristics	Relative WBP2 expression		*P*value
	Low (*n* = 24)	High (51)	
Gender			0.3289
Male	10	28	
Female	14	23	
Age			0.7765
≤60	17	39	
>60	7	12	
Tumor grade (IDH1/2 mutant) normal	2 (0)	1 (0)	0.0029**
G1	5 (1)	1 (0)	
G2	7 (4)	8 (3)	
G3 Tumor grade excluding IDH1/2 mutant Normal G1 G2 G3	10 (7) 2 4 3 3	41 (5) 1 1 5 36	0.0003**
Pathological type			0.3508
Pilocytic astrocytoma	1	1	
Diffuse astrocytoma anaplastic astrocytoma oligoastrocytomas glioblastoma	5 3 1 12	6 14 7 22	

### Upregulation of WBP2 induces cell overproliferation and cell cycle arrest

**Fig. 2:**
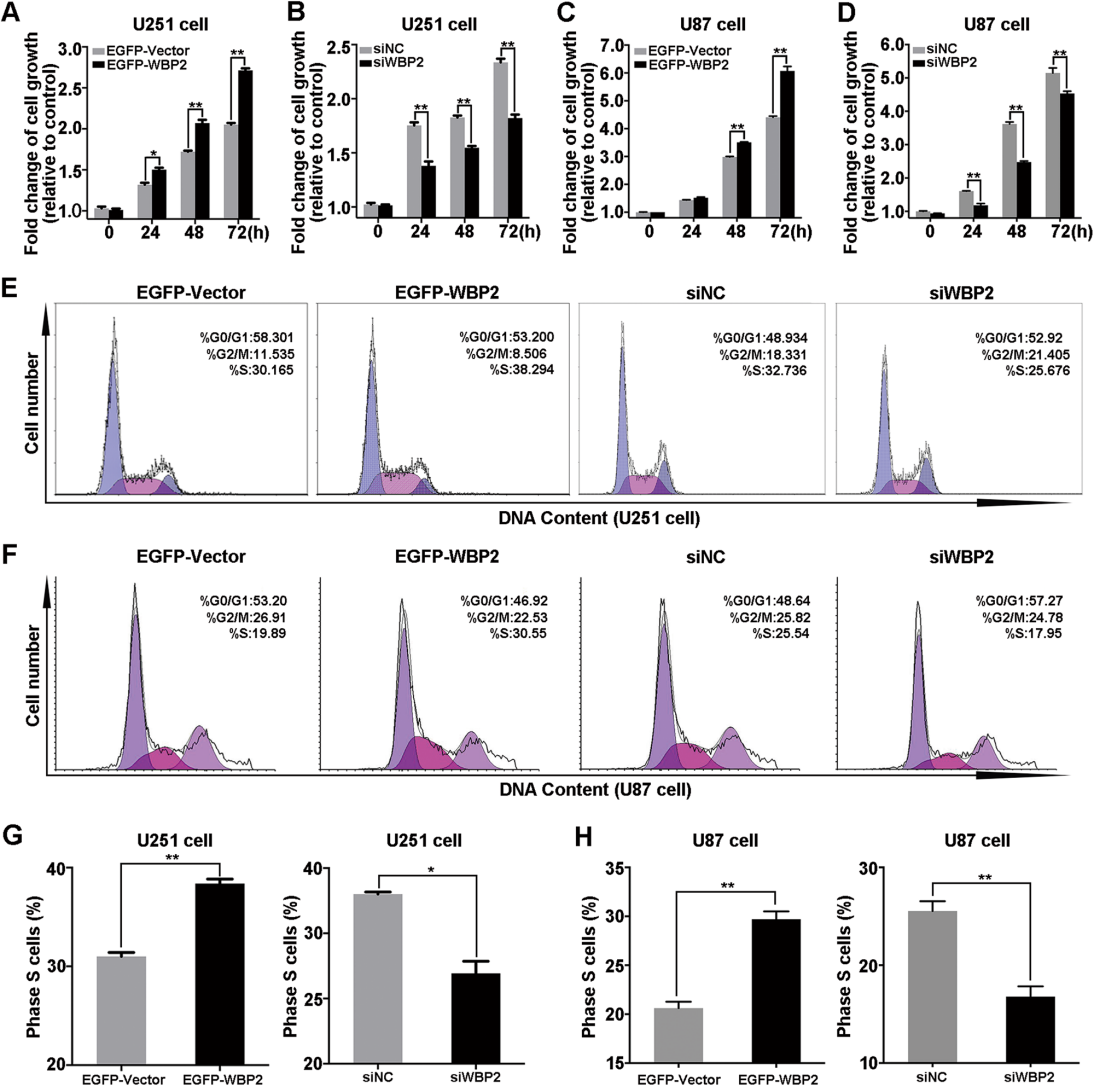
Effects of WBP2 on cell growth and cell cycle progress of U251. **a–d** After seeding for 24 h, U251 and U87 cells (eight groups of cells) were processed with MTT assay (EGFP-Vector, EGFP-WBP2, siNC, and siWBP2) at 0, 24, 48, and 72 h. **e** Cell cycle analysis of U251 cells transfected with EGFP-WBP2, EGFP-Vector, siNC and siWBP2. **f** Cell cycle analysis of U87 cells transfected with EGFP-WBP2, EGFP-Vector, siNC and siWBP2. **g**,** h** Representative quantity of cell numbers in S-phase in the eight group cells. EGFP-Vector, control stable cell line transfected with pEGFP-C1 plasmid in U251 or U87 cells; pEGFP-WBP2, stable cell lines transfected with pEGFP-WBP2; siNC, cells transfected with negative control siRNA; siWBP2, downregulation of WBP2 expression transfected with siRNA of WBP2 in U251 or U87 cells. *P < 0.05, **P < 0.01

To evaluate the potential impact of WBP2 on cell growth in glioma cells, we induced stable overexpression of WBP2 in U251 and U87 cells (EGFP-Vector and EGFP-WBP2 cell lines). We ablated the expression of WBP2 using RNAi-mediated knockdown in U251 and U87 cells to better interpret the role of WBP2 in the development of glioma. 3-(4,5-dimethylthiazol-2-yl)-2,5-diphenyltetrazo-liumbromide (MTT) assay results suggested that upregulation of WBP2 enhanced cell proliferation of glioma cells (Fig. 2a–d), while depletion of WBP2 inhibited cell proliferation. To investigate possible influencing factors in WBP2-mediated enhancement of glioma cell growth, we analyzed the cell cycle profile in WBP2-overexpressed and WBP2-knockdown cells by flow cytometry. Interestingly, stable expression of WBP2 cells significantly increased the S-phase population and decreased the cell population in the G0/G1 phase, indicating S-phase arrest induction by WBP2 overexpression compared with control cells (38.294% vs. 30.165% in U251 cells, 29.72% vs. 20.62% in U87 cells, *P* < 0.01) (Fig. 2e–h). However, when WBP2 expression was silenced in U251 and U87 cells, the proportion of S-phase cells was significantly decreased (25.676% vs. 32.736%, *P* < 0.05; 25.543% vs. 16.786%, *P* < 0.01), suggesting downregulation of WBP2 negatively modulated cell proliferation of U251 cells by affecting the number of S-phase cells (Fig. 2e–h). These results explained the impact of WBP2 on cell growth rates and the cell cycle period of glioma cells.

### WBP2 affects cell migration and tumorigenic ability in U251 cells

**Fig. 3:**
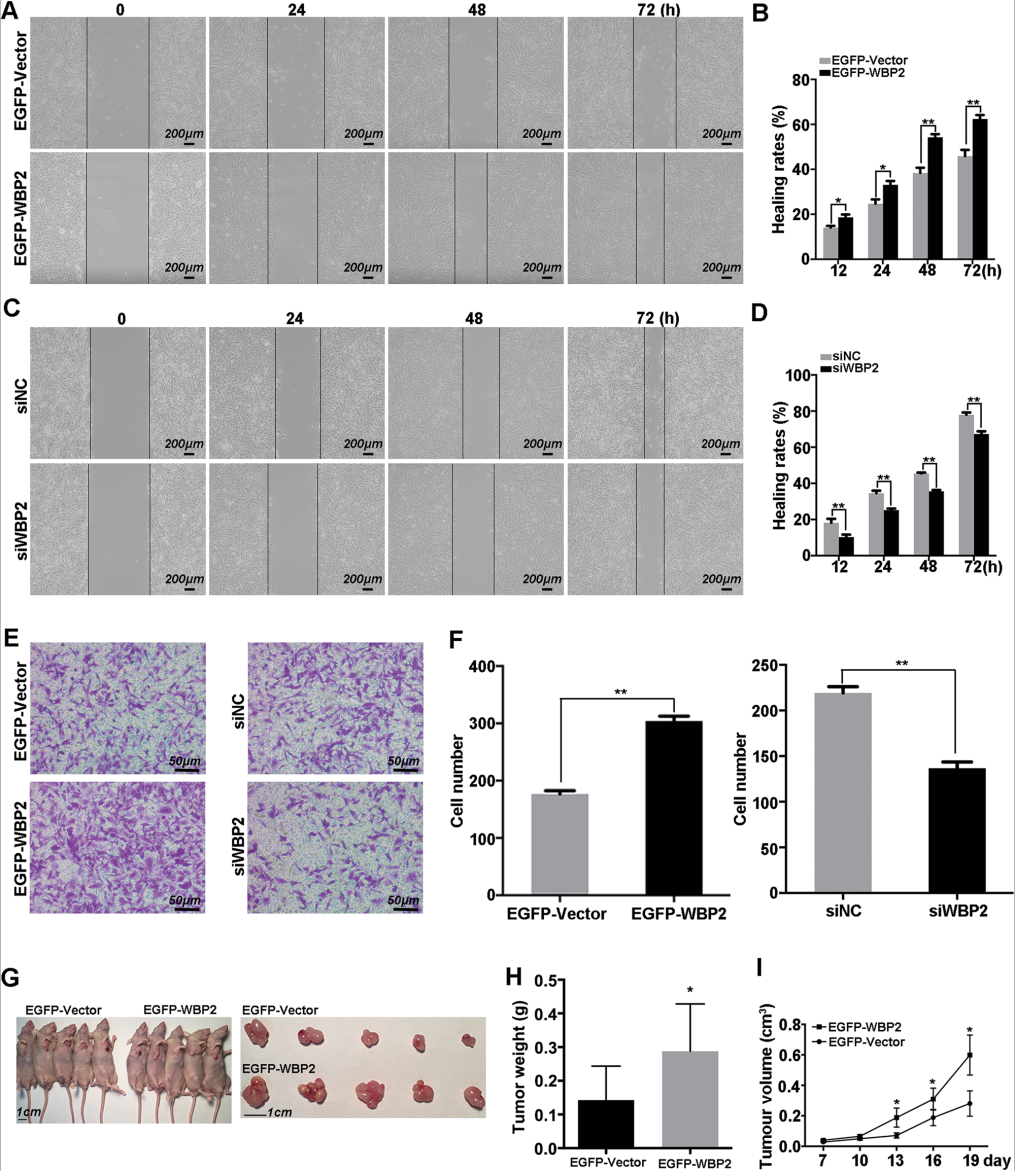
WBP2 regulates the migration ability of U251. **a** Photos of the wounded cells were taken at 0, 24, 48, 72 h after wound induction in EGFP-Vector and EGFP-WBP2 group cells. Scale bar, 200 μm. **b** Quantitative analysis of the healing rates of EGFP-Vector and EGFP-WBP2 group cells. **c** Images from the wound-healing experiment of the U251 cells after transient downregulation of WBP2. Scale bar, 200 μm. **d** Quantitative analysis of the healing rates of U251-siNC and U251-siWBP2 group cells. **e** Efficiency of WBP2 on the migration ability in U251 cells was determined by transwell assay. Scale bar, 50 μm. **f** Cells on the lower surface of the membrane quantified and analyzed with five randomly selected fields. **g** Images of EGFP-Vector and EGFP-WBP2 group cells from mice and mice tumor tissue (*n* = 5/group). Scale bar, 1 cm. **h** Tumor weight of EGFP-Vector and EGFP-WBP2 cells. **i** The tumor growth curves of the EGFP-Vector and EGFP-WBP2 groups. *P < 0.05, **P < 0.01

WBP2 has been reported to play a critical role in promoting cell metastasis in breast cancer cells^22^. To address the role of WBP2 on cell transferability in U251 cells, we performed wound-healing and transwell assays to determine the relationship between them in four different cell lines. For wound-healing assay, pictures were taken promptly at fixed intervals after wound induction. As shown in Fig. 3a, overexpression of WBP2 significantly enhanced wound-healing abilities compared with control group cells. The healing rates were also significantly higher in EGFP-WBP2 cells than in control cells (Fig. 3b). Conversely, the healing capability of WBP2-silenced cells was notably improved compared with control cells (Fig. 3c, d). We also performed transwell assay to confirm this phenotype, and the results indicated that WBP2 overexpression promoted, whereas WBP2 silencing restrained, the number of migration cells in U251 cells (Fig. 3e, f). Additionally, transwell assay using U87 cells also showed a promotion effect on migration ability under the condition of WBP2 upregulation, but migration rate of U87 cells was vividly restrained in absence of WBP2 (Fig. S2A-B). To ascertain the impact of WBP2 on *in vivo* tumorigenicity of U251 cells, we analyzed the tumor parameters in the control and experimental groups. For cells transfected with EGFP-WBP2, tumor size and tumor weight was greater than in the control group (Fig. 3g–f). WBP2 upregulation obviously promoted tumor growth in comparison with the control group (Fig. 3i). These findings hint that WBP2 positively regulates the metastasis and tumorigenesis of glioma cells.

### WBP2 directly interacts with ENO1 and Homer3 in U251 cells

**Fig. 4:**
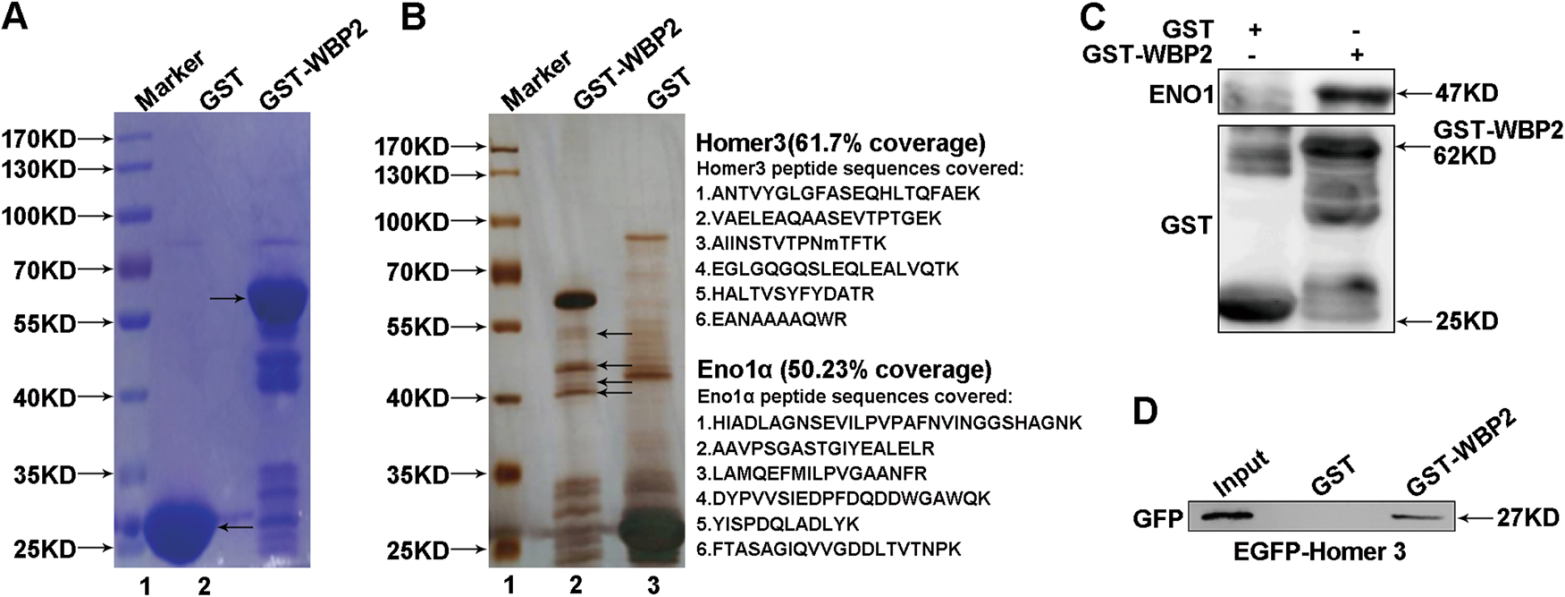
WBP2 directly binds to ENO1 and Homer3 in U251 cells . **a** Recombinant human GST-WBP2 protein was purified by adsorption on L-glutathione-sepharose. **b** SDS-PAGE showed several proteins identified by GST large-scale pulldown. **c**, **d** Exogenous ENO1 and Homer3 transfected in U251 cells binds to GST-WBP2 fusion protein, as revealed by GST pulldown using immobilized GST-WBP2

As indicated above, WBP2 is characterized as a particular cancer-promoting feature of glioma cells. To determine the underlying molecular mechanisms by which WBP2 regulates glioma cell properties, we examined the protein partners of human WBP2 in glioma cells by affinity adsorption using the plasmid pGEX-4T-1-GST-WBP2. We then used the recombinant protein GST-WBP2 as an acceptor to incubate the U251 cells with lysates (Fig. 4a), and the extract incubated with the GST protein under the same conditions served as a negative control. Several different bands were separated by SDS-PAGE and potential proteins precipitated with the GST-WBP2 by MS were identified (Fig. 4b). Results showed that NEDD4 and YAP1 possessed relatively high coverage of the polypeptide and peptide sequences with WBP2; this was reported by others a few decades ago^23,24^, and reinforces the authenticity of our work (Table 2). We screened two potential partner proteins, ENO1 and Homer3, that showed high coverage with WBP2; their corresponding genes are vital in the development of glioma and the nervous system, respectively. To verify the MS result, we used purified L-glutathione–sepharose-immobilized GST-WBP2 protein incubated with ENO1 and Homer3-overexpressed cell lysates to confirm the direct interaction of ENO1 and Homer3 in U251 cells. The results showed that both ENO1 and Homer3 combined with WBP2 directly in vitro revealing these two interacting partners of WBP2 for the first time (Fig. 4c-d). Their interaction is probably part of the mechanism by which WBP2 regulates the proliferation and migration of glioma cells.

**Table 2:** Potential interacting protein partners of the human protein WBP2

Accession	Gene	Score	Coverage	MW [kDa]	Disease	Reference
38605068	Homer3	172	61.77	39.8	Neuronal signaling (20)	This study
P68104	ENO1α	55	50.23	47.1	Lymphocytic hypophysitis, autoimmune retinopathy	This study
P50552	VASP	198.35	41.84	39.8	Immune System	This study
P14136-3	GFAP	67.6	51.51	49.5	Alexander disease	This study
55977767	VIM	46.5	34.9	53.6	Peritoneal mesothelioma	This study
P07437	Tubulinβ	193.4	51.8	49.6	Brain malformations	This study
Q96J02-2	ITCH	40.9	26.2	98.6	Autoimmune disease	This study
P46934-4	NEDD4	20.41	16	104.2	Liddle syndrome	(23)
E9PRV2	YAP1	6.77	9.56	48.2	Intellectual disability	(24)

### Removal of ENO1 and Homer3 suppresses tumor-promoting action evoked by WBP2-overexpression

**Fig. 5:**
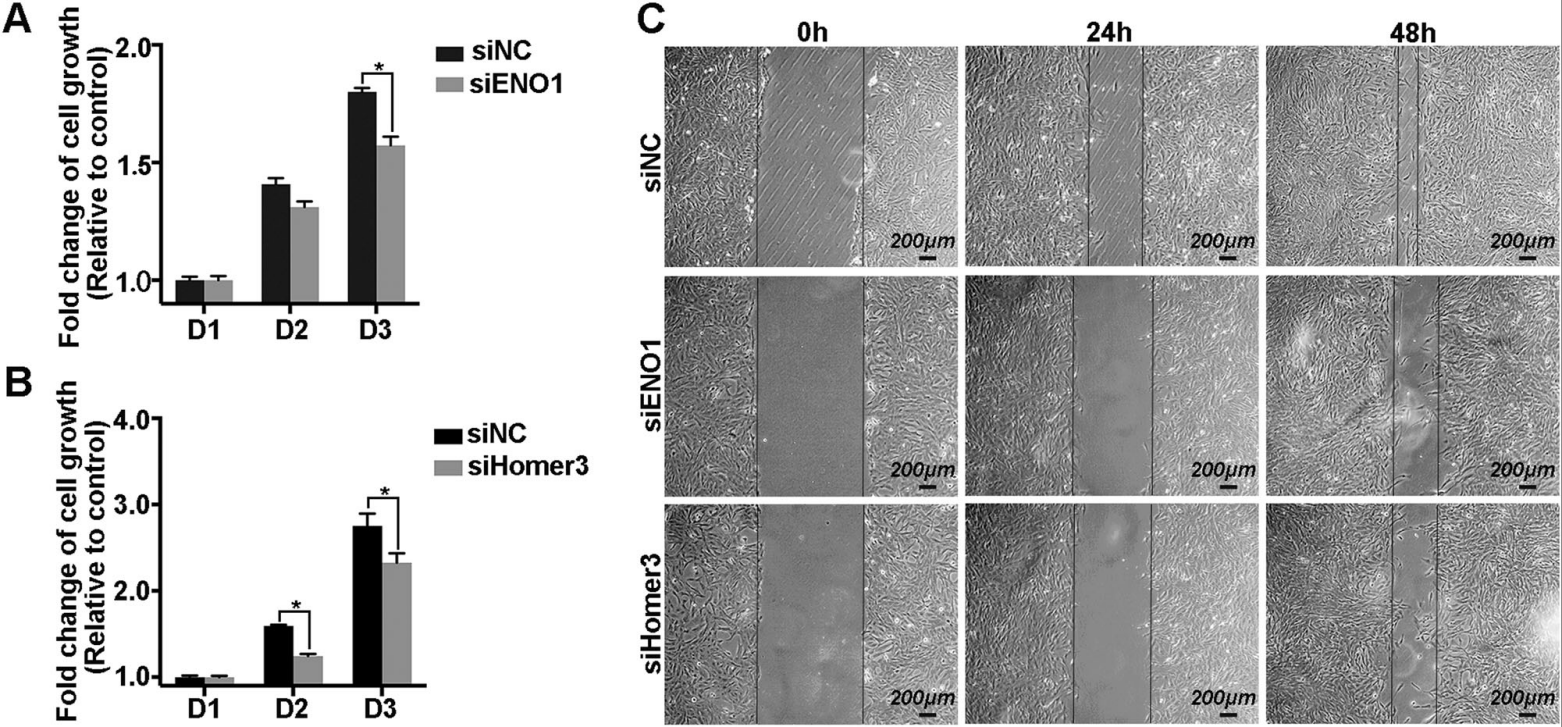
Transiently suppressed ENO1 and Homer3 inhibits WBP2-mediated U251 cell overgrowth and metastasis. **a** and **b** MTT assay was performed to test the effect of ENO1 and Homer3 silenced by the increased cell proliferation ability induced by WBP2 overexpression. **c** Ablation of ENO1 and Homer3 reduced the healing rates in EGFP-WBP2 group cells. Scale bar, 200 μm. siNC, cells transfected with negative control siRNA; siENO1, cells transfected with ENO1-siRNA; siHomer3, cells transfected with Homer3-siRNA. *P < 0.05

To explore whether ENO1 and Homer3 were accomplices of WBP2 in carcinogenesis, we conducted MTT and wound-healing experiments with depletion of ENO1 or Homer3 in WBP2-overexpressed cells. The rates of cell proliferation were notably faster in ENO1-silenced or Homer3-silenced EGFP-WBP2 group cells than in the EGFP-WBP2 group cells not silenced (Fig. 5a-b). Knockdown of ENO1 and Homer3 in EGFP-WBP2 group cells markedly decreased healing speed compared with EGFP-WBP2 cells transfected with negative control siRNA (Fig. 5c). Therefore, our data confirm that interactions between ENO1, Homer3, and WBP2 potentially mediate the cancer-promoting effects initiated by upregulation of WBP2 in glioma cells.

### WBP2 Regulates the expression of ENO1 and the activation of Akt

**Fig. 6:**
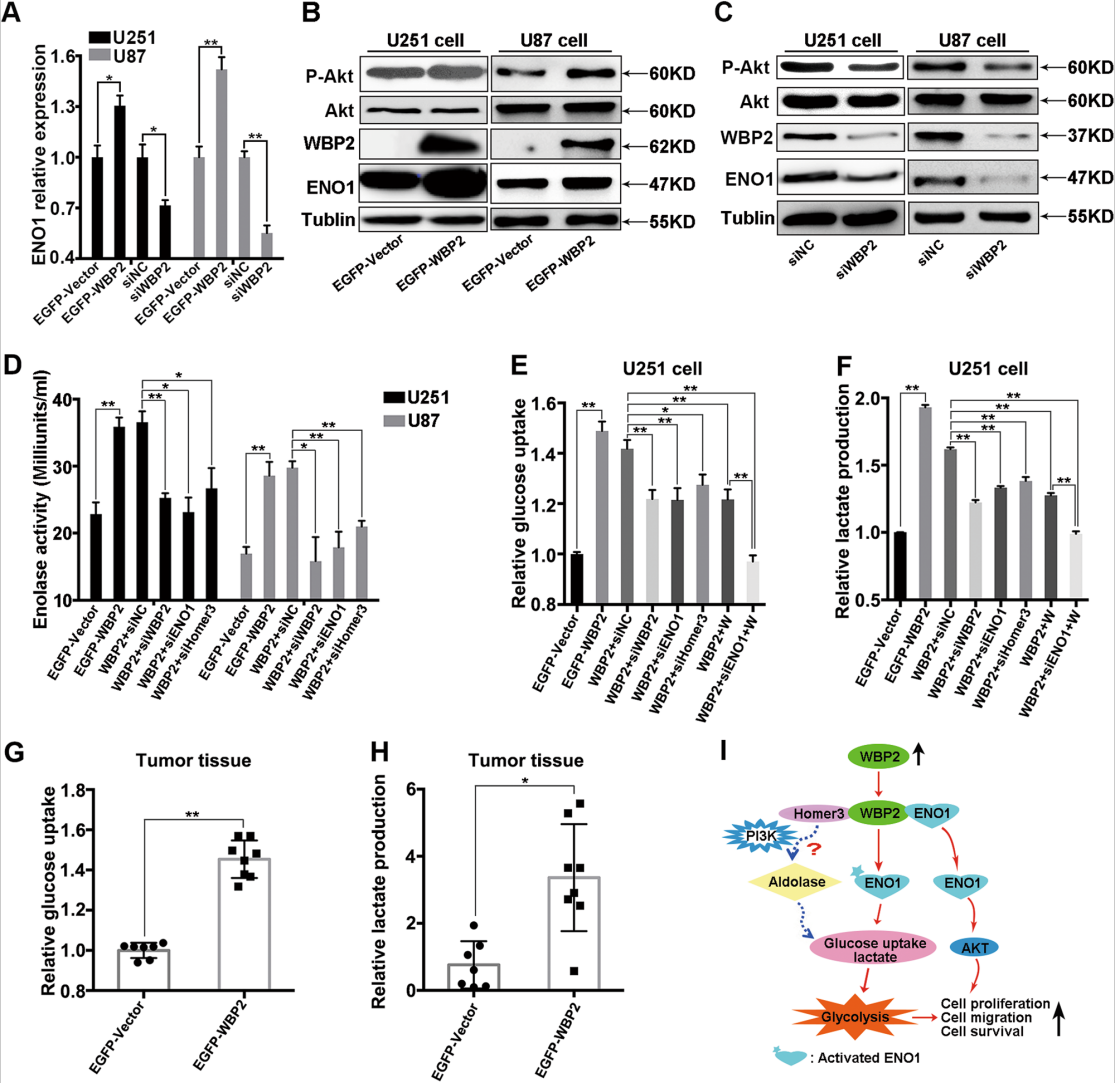
WBP2 affects the expression and activity of ENO1 in glioma cells. **a** WBP2 affects the transcription of *ENO1* in U251 and U87 cells. **b**, **c** Phosphorylation of Akt and expression of ENO1 measured by western blot using specific antibodies in four cell groups. **d** Measurements of Enolase-1 activity in different group cells, following an enolase activity assay, **e**, **f** Relative glucose uptake and lactate production of eight group cells, assessed by their corresponding detection kits. **g**, **h** Relative glucose uptake and lactate production in xenografted tumor. **i** The proposed model of WBP2-mediated cellular processes in Glioma cells. Tubulin served as the internal control. WBP2 + siNC, EGFP-WBP2 group cells transfected with negative control siRNA; WBP2 + siWBP2, EGFP-WBP2 group cells transfected with WBP2-siRNA; WBP2 + siENO1, EGFP-WBP2 group cells transfected with ENO1-siRNA; WBP2 + siHomer3, EGFP-WBP2 group cells transfected with Homer3-siRNA; WBP2 + W, EGFP-WBP2 group cells treated with wortmannin; WBP2 + siENO1 + W, EGFP-WBP2 group cells transfected with ENO1 siRNA that were pretreated with wortmannin. *P < 0.05, **P < 0.01

Having shown that ENO1 and Homer3 mediated cancer cell growth and metastasis induced by WBP2 overexpression, we explored the underlying mechanism of WBP2 acting as a key regulator of tumor development. We found that the transcription of *ENO1* was upregulated in the EGFP-WBP2 group and downregulated in the WBP2-silenced glioma cells (Fig. 6a). Similarly, there was a dramatic increase in ENO1 protein levels accompanying the upregulation of WBP2, whereas WBP2 expression was significantly inhibited when endogenous WBP2 was removed from U251 and U87 cells (Fig. 6b, c, second band from the bottom). Previous research has indicated that ENO1 can positively affect the PI3K/Akt signaling pathway, promoting cell growth and migration in glioma cell^6^. We assessed changes in PI3K/Akt signaling under conditions of WBP2 overexpression and WBP2 silencing in glioma cells. Similar with the results for ENO1, phosphorylation of Akt was expedited by overexpression of WBP2 in U251 and U87 cells (Fig. 6b). Conversely, Akt activity was suppressed with knockdown of WBP2 expression (Fig. 6c, first band). Moreover, we determined the influence of ENO1 level on the WBP2 expression and its downstream signaling pathway. Knockdown of ENO-1 did not change the expression of WBP2, c-Myc, and phosphorylated NF-kB, but inhibited the expression of N-cadherin, phosphorylated ERK1/2, phosphorylated p38, and phosphorylated Akt accompanied by the increased E-cadherin (Fig. S3). These results suggest that WBP2 potentially mediates gliomagenesis by modulating ENO1 expression and its downstream signaling activity.

### ENO1/Akt signaling pathway is required for WBP2-mediated Increase in glycolytic activity

ENO1 is a glycolytic enzyme expressed in most tissues and malignant cells, in which active glycolysis provides energy for cancer cell metabolism^25^. The PI3K-Akt signaling pathway drives cancer cells to favor glycolysis over mitochondrial oxidation^26^. To explore the correlation between WBP2 and ENO1 activity, we determined the ENO1 values in six groups of glioma cells (EGFP-Vector, EGFP-WBP2, WBP2 + siNC, WBP2 + siENO1, WBP2 + siWBP2, and WBP2 + siHomer3). The serial enolase-1 concentrations were significantly upregulated along with an increase in WBP2 expression, but ENO1 activity was notably reduced after transfection with siWBP2 in cells with WBP2 overexpression (Fig. 6d). Simultaneously, we detected the ENO1 values in WBP2 + siENO1 and WBP2 + siHomer3 cells, showing that ablation of ENO1 in the EGFP-WBP2 group returned ENO1 concentrations to normal levels (Fig. 6d). Additionally, we evaluated the glucose uptake and lactate production of U251 cells under different conditions. Compared with the EGFP-Vector control group, WBP2 overexpression obviously promoted glucose consumption and lactate formation in glioma cells (Fig. 6e-f, left two bars). However, when WBP2, ENO1, and Homer3 were downregulated using RNAi-mediated knockdown in cells transfected with WBP2, absorption of glucose and secretion of lactate were significantly suppressed in comparison with WBP2-overexpressed cells transfected with negative control siRNA (middle four bars). For further investigation, we blocked the PI3K/Akt signaling pathway using wortmannin (a specific inhibitor of PI3K) to confirm whether the ENO1/Akt signaling pathway was involved in the glycolytic cycle in WBP2-overexpressed cells. Our findings indicated that relative glucose and lactate production of U251 cells was repressed when cells were pretreated with wortmannin, and they were further significantly inhibited by wortmannin treatment after transfection with the siRNA of ENO1. This suggests that ENO1/Akt is the key signaling axis in the WBP2-mediated increase in glucose uptake and lactate secretion.

We also detected the absorption rate of glucose and amount of secreted lactate in the tumor tissues of mice. Both glucose uptake and lactate production were robustly enhanced in the presence of exogenous WBP2, compared with the negative control (EGFP-Vector group) (Fig. 6g-h). In short, our findings illustrate that WBP2 modulates the expression and activity of ENO1 and further mediates EMP pathway activity through the PI3K/Akt signaling pathway, to regulate the biologic behavior of glioma cells.

## Discussion

WBP2 is widely expressed in most tissues and has been identified as being involved in protein–protein interactions via its PY motifs in the C-terminal region^27^. As a binding partner of WW domain proteins, it can be coupled with yes-associated protein (Yap), E3 ubiquitin-protein ligase RSP5 (Rsp-5), Amyloid beta A4 precursor protein-binding family B member 1 (FE65), neural precursor cell expressed developmentally down-regulated protein 4 (Nedd4), and WW domain-containing transcription regulator protein 1 (TAZ), which are all WW domain-containing proteins^22^. WBP2 also combines with Paired box (Pax) and E6AP ubiquitin-protein ligase (E6-AP), which do not contain WW domain in mammals and function as an adapter molecule^13,28^. Most studies of WBP2 have focused on its function as a coactivator of ER, and its role in promoting tumor cell proliferation in ER-positive tissue or cell lines.

In the present study, we discovered a high expression of WBP2 in several tumor cell lines and glioma tissue chip. We also found that overexpression of WBP2 enhanced, while downregulation of WBP2 expression suppressed, the proliferation, migration, and tumor formation ability of Glioma cells. To assess whether WBP2 was involved in the progression of glioma, we performed pulldown/MS analysis using recombinant GST-WBP2 protein as a bait to screen for partners of WBP2 in U251 cells, identifying some potential proteins precipitated with GST-WBP2 (Table 1). Our data indicated that ENO1 and Homer3 had high coverage rates with WBP2, and previous studies have confirmed that both ENO1 and Homer3 are associated with glioma and nervous system development, suggesting these two proteins are an integral part in WBP2-mediated glioma cell growth and metastasis^6,29^.

Highly expressed ENO1 is correlated with non-Hodgkin lymphoma and glioma progression^6,7^. As a negative regulator of the PI3K/Akt pathway, depleted expression of ENO1 suppresses proliferation, metastasis, and invasive progression of glioma cells^6^. We found that forced expression of WBP2 promoted, while limited expression of WBP2 inhibited, ENO1 expression and its downstream PI3K/Akt signaling pathway in glioma cells. Our research illustrated a relationship between WBP2 and glycolysis enzyme activity in tumor cells and revealed a novel potential regulatory mechanism in glioma progression.

Homer3 has been shown to play a part in actin dynamics in neuronal stimulation^20,30^. Tumor cell dissemination and metastasis are largely launched by dynamic reorganization of the actin cytoskeleton^31–33^ and emerging research suggests that phosphoinositide 3-kinase regulates glycolysis, providing energy for tumor cells by mobilizing aldolase from the actin cytoskeleton^34^. This provides further evidence that Homer3, as a dendritic protein, potentially contributes to worsening glioma by affecting the glycolysis system (Fig. 6i). We determined that either knockdown of WBP2 or limiting the expression of Homer3 in stable cell lines with highly expressed WBP2 inhibited the activity of ENO1, indicating that WBP2 binding to Homer3 and ENO1 potentially promoted glioma cell growth and metastatic ability, possibly by acting on the glycolysis system.

Augmented glycolysis characterizes tumor cell metabolism^35^. In comparison with normal cells, tumor cells are preferentially dependent on aerobic glycolysis, which provides energy for glioma tumor cell metabolism by enhancing glucose uptake following an increase in lactate secretion^36–39^. We not only discovered that upregulation of WBP2 accelerated glucose uptake rate, but that it also enhanced the secretion speed of lactate in glioma cell lines and the tumor mass of mice. However, ablation of WBP2, ENO1, and Homer3 in cells transfected with exogenous WBP2 obviously inhibited WBP2 overexpression-mediated the increase in glycolysis. Pretreatment of cells with wortmannin achieved the same effect. These results verified our hypothesis, showing that the ENO1/Akt signaling pathway was important in WBP2-evoked enhancement of the pathway (EMP) (Fig. 6i).

In summary, we illustrated the carcinogenetic effect of WBP2 in glioma development. We also revealed how WBP2 promotes cell proliferation and regulates the cell cycle by modulating ENO1 activity and the ENO1-PI3K/Akt signaling pathway by binding to ENO1 and Homer3 in glioma cells. Further studies are needed to determine the precise mechanism by which WBP2 acts on a molecular level in the EMP pathway, indicating a novel therapeutic target for glioblastoma treatment.

## Materials and methods

### Materials

Primary antibodies against WBP2 (GTX81293) and Homer3 (GTX115242) were obtained from GeneTex. Primary antibody against IDH1/2 mutant (R132/172) was purchased from Meck Millipore (MABC1103). P-Akt^Tyr473^ (4060) was obtained from Cell Signaling Technology. Antibodies against Akt1/2/3 (sc-8312) and ENO1 (D121999) were purchased from Santa Cruz Biotechnology and Sangon Biotech, respectively. Wortmannin was purchased from Cell Signaling Technology.

### Cell culture, transfection and cell line construction

U251 and U87 cells were cultured in DMEM/F12 (Hyclone, USA) medium supplemented with 10% fetal calf serum (Hyclone) at 37 °C with 5% CO_2_. U251 cells were transfected with different plasmids using Lipofectamin™2000 (Thermo Fisher Scientific, US) according to the manufacturer’s instructions (Invitrogen, US). For EGFP-Vector and EGFP-WBP2 cell line construction, U251 and U87 cells were transfected with pEGFP-C1 and pEGFP-C1-WBP2 plasmids and cells stably expressing WBP2 were selected with medium containing G418. For RNAi-mediated knockdown of WBP2, cells were transfected with negative control and WBP2 siRNA (GAACUCACAUUCAAUGACA) and were used to protein and mRNA extraction in the cellular period 72 h after transfection.

### MTT assay

MTT assay was used to assess cell growth. U251 cells were seeded at a density of 3000 cells/well in a 96-well plate. The cells were incubated in culture media with 5 mg/mL MTT (Sigma) at 37 °C with 5% CO_2_ for 4 h. Following this, 150 μL DMSO was added to each well to dissolve the cells. Samples were collected at days 1, 2, 3, and 4, and were measured at a wavelength of 492 nm using ultraviolet spectrophotometer.

### Migration assay

A wound-healing assay was performed with cells grown to 90% confluence and an injury line made using a 2-mm-wide plastic pipette tip. The excess liquid in the wells was removed, and the wells rinsed with phosphate-buffered saline and cultured with serum-free DMEM/F12 medium. The cells were incubated for 60 h to allow cell growth. The cell migration area was examined using an inverted microscope (Zeiss, Germany) and then photographed at different time points. The migration ability of cells was assessed by transwell assay in 24-well plates (Corning, USA). Cells at the logarithmic phase were digested with 0.25% trypsin and washed twice with 1 × PBS. A total of 1 × 10^5^ cells/100 μL were counted and inoculated into the upper chamber of the 24-well plate. A volume of 600 μL of DMEM/F12 medium containing 10% fetal bovine serum was added to the lower chamber for incubation at 37 °C in 5% CO_2_ for 24 h. The upper chamber was removed and washed twice with 1× PBS to remove the non-migrated cells from the upper surface of the membrane. The migrated cells on the bottom surface were stained with 0.1% crystal violet and the number of cells then counted. Data were obtained from three independent experiments.

### Cell cycle analysis

The cell density was adjusted to 1 × 10^6^ cells/mL and the cells were digested with 0.05% trypsin. Detached cells were washed with 1 × PBS twice and the detached cells were resuspended in 70% cold ethanol and incubated overnight at 4 °C. After removal from the ethanol, cells were resuspended in PBS (10^6^ cells/100 µL), then incubated with 50 μg/mL RNA enzyme and 1 μg/mL propidium iodide in the dark for 30 min. The labeled cells were analyzed using flow cytometry (Beckman–Coulter, US).

### GST pulldown and coimmunoprecipitation assay

GST pulldown was performed using the expression of WBP2 in *Escherichia coli* BL21 as a GST-fusion protein. Briefly, *E. coli* BL21 were grown overnight (12 h), 100-fold diluted in fresh Lysogeny broth with 100 mg/L ampicillin, and incubated at 37 °C until an OD600 of 0.4–0.6 was reached. Expression of the recombinant protein was induced with 0.1 mM isopropyl-β-d-thiogalactoside for 12 h at 24 °C. The protein was then purified and immobilized on glutathione–agarose (Sigma–Aldrich, Germany) and the immobilized GST-fusion proteins were run on SDS-PAGE to evaluate the amount of each protein. For the coimmunoprecipitation experiment, similar amounts of GST-WBP2 and GST-alone (as negative control) were incubated with 30 mg of U251 lysates overnight at 4 °C, to determine any binding interactions. After removing the supernatant, the resin was washed three times with lysis buffer. The proteins were analyzed using SDS-PAGE and targeted with specific primary antibody.

### Mass spectrometry

The trypsin digestions of GST-WBP2 pulldown elutions were analyzed by LC-MS/MS and high resolution accurate mass data were acquired in both positive and negative ion mode using a Q Exactive Orbitrap mass spectrometer (Thermo Scientific).

### Western blot analysis

Protein samples from different cells were extracted for western blot analysis, separated by SDS-PAGE and immunoblotted using the specific primary antibodies. The membrane was then incubated with HRP-conjugated anti-rabbit or anti-mouse IgG secondary antibody (Thermo Fisher Scientific). Protein bands were detected using Western Bright ECL (Advansta, US) and autoradiography performed. Tubulin and Gapdh were used as the internal controls.

### RNA extraction and quantitative real-time PCR analysis

Total RNA was extracted from cells using Trizol reagent (Invitrogen), and the cDNA synthesized using First Strand cDNA Synthesis Kit (Transgene, Beijing, China) according to the manufacturer’s instructions. The cDNA obtained was used for quantitative real-time PCR with an Mx3005p system (Stratagene, US). The primer sequences used in the study are shown in Table S1. PCR conditions used were 95 °C for 10 min to activate DNA polymerase, followed by 40 cycles of 90 °C for 15 s, 60 °C for 1 min. Relative gene expression was analyzed using the 2-∆∆Ct method, and each sample was tested in triplicate. Gene expression was normalized to *GAPDH*.

### Xenograft experiment

Male nude mice (5–6 weeks old, *n* = 5/group) were randomly divided into two groups. The mice were housed five per cage in a room under controlled light (12 h/day) and temperature (22 ± 2 °C) conditions. After a week of adjustable feeding, the mice were subcutaneously injected with 2 × 10^6^ cells (EGFP-Vector and EGFP-WBP2 cells). The resulting tumors were measured at day 6 after initial injection and every 4 days after. At day 30, the tumors were exposed, photographed and weighed. Tumor volume was calculated as 0.5 × length × width × width. All experimental procedures were approved by the Animal Welfare Committee of Research Organization (X201011), Xiamen University.

### Enolase activity

Enolase activity was determined using Enolase Activity Assay Kit according to the manufacturer’s instruction (Sigma, Germany, MAK178-1KT). In brief, the samples of cell lysates were mixed with reaction buffer and incubated at 25 °C. After 5–10 min, an initial measurement of OD value was taken at a wavelength of 570 nm, and was followed by measurement every 2–3 min until the OD value of the most active sample was greater than the value of the highest standard for getting the final measurement. Enolase activity was calculated using an equation described previously^40^.

### Measurement of glucose and lactate

For the detection of glucose uptake and lactate production, cells were divided into eight groups (EGFP-Vector, EGFP-WBP2, WBP2 + siNC, WBP2 + siWBP2, WBP2 + siENO1, WBP2 + siHomer3, WBP2 + W, WBP2 + siENO1 + W). After transfection for 48 h with specific siRNA, phenol red free medium was used to culture cells for 24 h and the glucose consumption and lactate formation were measured using Glucose Assay Kit and Lactic Acid Kit (Jiancheng Bioengineering Institute, China) according to the manufacturer’s instructions. For inhibitor assay, cells were pretreated with wortmannin (1 μM) for 2 h after transfection for 48 h; glucose and lactate were then measured as described above. For the tumor tissues, glucose uptake and lactate secretion were detected using the same kits, after homogenization.

### Immunohistochemistry staining

Tissue microarray of Glioma was purchased from Shanghai Outdo Biotech Co (Shanghai, China). In brief, the tissue microarray was dewaxed and rehydrated with alcohol gradient. After antigen retrieval with citrate buffer for 5 min, paraffin sections were incubated with 3% H_2_O_2_ for 20 min and then blocked with 1% BSA for 1 h. Primary antibody against WBP2 was used to detect the level of expression of WBP2 protein in glioma tissue from patients. The staining result was analyzed with microscopy.

### Statistical analysis

Data were analyzed for three independent experiments and GraphPad Prism 5.0 (GraphPad Software Inc, USA) was used for statistical analysis. The results were presented as mean ± standard error of the mean. Statistical comparisons were performed using Student’s t-test or one-way analysis of variance and significance accepted at *P* < 0.05.

## Electronic supplementary material

SupplementaryFigures

## References

[1] Yi GZ (2016). Akt and β-catenin contribute to TMZ resistance and EMT of MGMT negative malignant glioma cell line. J. Neurol. Sci..

[2] Sakr M (2016). miR-150-5p and miR-133a suppress glioma cell proliferation and migration through targeting membrane-type-1 matrix metalloproteinase. Gene.

[3] Peng Z (2016). MicroRNA-370-3p inhibits human glioma cell proliferation and induces cell cycle arrest by directly targeting β-catenin. Brain Res..

[4] Porporato PE (2014). A mitochondrial switch promotes tumor metastasis. Cell Rep..

[5] Poteet E (2013). Reversing the Warburg effect as a treatment for glioblastoma. J. Biol. Chem..

[6] Song Y (2014). Alpha-enolase as a potential cancer prognostic marker promotes cell growth, migration, and invasion in glioma. Mol. Cancer.

[7] Zhu X (2015). ENO1 promotes tumor proliferation and cell adhesion mediated drug resistance (CAM-DR) in Non-Hodgkin’s Lymphomas. Exp. Cell Res..

[8] Hsiao KC (2013). Surface α-enolase promotes extracellular matrix degradation and tumor metastasis and represents a new therapeutic target. Plos One.

[9] Chen S, Duan G, Zhang R, Fan Q (2014). Helicobacter pylori cytotoxin-associated gene A protein upregulates α-enolase expression via Src/MEK/ERK pathway: implication for progression of gastric cancer. Int. J. Oncol..

[10] Gao J (2013). Role of enolase-1 in response to hypoxia in breast cancer: exploring the mechanisms of action. Oncol. Rep..

[11] Macias MJ, Wiesner S, Sudol M (2002). WW and SH3 domains, two different scaffolds to recognize proline-rich ligands. FEBS Lett..

[12] Chen Y (2007). Differential expression of novel tyrosine kinase substrates during breast cancer development. Mol. Cell Proteom..

[13] Dhananjayan SC (2006). WW domain binding protein-2, an E6-associated protein interacting protein, acts as a coactivator of estrogen and progesterone receptors. Mol. Endocrinol..

[14] Lim SK, Orhant-Prioux M, Toy W, Tan KY, Lim YP (2011). Tyrosine phosphorylation of transcriptional coactivator WW-domain binding protein 2 regulates estrogen receptor α function in breast cancer via the Wnt pathway. FASEB J..

[15] Buniello A (2016). Wbp2 is required for normal glutamatergic synapses in the cochlea and is crucial for hearing. EMBO Mol. Med..

[16] Fornage M (2011). Genome-wide association studies of cerebral white matter lesion burden: the CHARGE consortium. Ann. Neurol..

[17] Lim SK (2016). Wnt signaling promotes breast cancer by blocking ITCH-mediated degradation of the YAP/TAZ transcriptional coactivator WBP2. Cancer Res..

[18] Chen S (2017). WW domain-binding protein 2: an adaptor protein closely linked to the development of breast cancer. Mol. Cancer.

[19] Shiraishi Y, Mizutani A, Yuasa S, Mikoshiba K, Furuichi T (2004). Differential expression of Homer family proteins in the developing mouse brain. J. Comp. Neurol..

[20] Shiraishi-Yamaguchi Y, Furuichi T (2007). The Homer family proteins. Genome Biol..

[21] Nourashrafeddin S (2015). Elevated expression of the testis-specific gene WBP2NL in breast cancer. Biomark. Cancer.

[22] Chan SW (2011). WW domain-mediated interaction with Wbp2 is important for the oncogenic property of TAZ. Oncogene.

[23] Van Huysse JW, Amin MS, Yang B, Leenen FHH (2012). Salt-induced hypertension in a mouse model of Liddle syndrome is mediated by epithelial sodium channels in the brain. Hypertension.

[24] Williamson KA (2014). Heterozygous loss-of-function mutations in YAP1 cause both isolated and syndromic optic fissure closure defects. Am. J. Hum. Genet..

[25] Gill KS (2016). Glycolysis inhibition as a cancer treatment and its role in an anti-tumour immune response. Biochim. Biophys. Acta.

[26] Yu L, Chen X, Wang L, Chen S (2016). The sweet trap in tumors: aerobic glycolysis and potential targets for therapy. Oncotarget.

[27] Chen HI, Sudol M (1995). The WW domain of Yes-associated protein binds a proline-rich ligand that differs from the consensus established for Src homology 3-binding modules. Proc. Natl. Acad. Sci. USA.

[28] Nitsch R, Di Palma T, Mascia A, Zannini M (2004). WBP-2, a WW domain binding protein, interacts with the thyroid-specific transcription factor Pax8. Biochem. J..

[29] Foa L, Gasperini R (2009). Developmental roles for Homer: more than just a pretty scaffold. J. Neurochem..

[30] Shiraishi-Yamaguchi Y (2009). Interaction of Cupidin/Homer2 with two actin cytoskeletal regulators, Cdc42 small GTPase and Drebrin, in dendritic spines. BMC Neurosci..

[31] Palmer TD, Ashby WJ, Lewis JD, Zijlstra A (2011). Targeting tumor cell motility to prevent metastasis. Adv. Drug Deliv. Rev..

[32] Yamaguchi H, Condeelis J (2007). Regulation of the actin cytoskeleton in cancer cell migration and invasion. Biochim. Biophys. Acta.

[33] McSherry EA, Donatello S, Hopkins AM, McDonnell S (2007). Molecular basis of invasion in breast cancer. Cell Mol. Life Sci..

[34] Hu H (2016). Phosphoinositide 3-kinase regulates glycolysis through mobilization of aldolase from the actin cytoskeleton. Cell.

[35] Gabriely G, Wheeler MA, Takenaka MC, Quintana FJ (2017). Role of AHR and HIF-1α in glioblastoma metabolism. Trends Endocrinol. Metab..

[36] Balaban RS (1990). Regulation of oxidative phosphorylation in the mammalian cell. Am. J. Physiol..

[37] Cairns RA, Harris IS, Mak TW (2011). Regulation of cancer cell metabolism. Nat. Rev. Cancer.

[38] Lunt SY, Vander Heiden MG (2011). Aerobic glycolysis: meeting the metabolic requirements of cell proliferation. Annu. Rev. Cell Dev. Biol..

[39] Jensen RL (2009). Brain tumor hypoxia: tumorigenesis, angiogenesis, imaging, pseudoprogression, and as a therapeutic target. J. Neurooncol..

[40] Pancholi V (2001). Multifunctional alpha-enolase: its role in diseases. Cell Mol. Life Sci..

