# Supplementary material for: WW domain-binding protein 2 acts as an oncogene by modulating the activity of the glycolytic enzyme ENO1 in glioma
Source: Cell Death Dis. 2018 Mar 1;9(3):347. doi: 10.1038/s41419-018-0376-5 (PMC5832848; doi:10.1038/s41419-018-0376-5)
Supplement: Supplementary file 1 — SupplementaryFigures [file 41419_2018_376_MOESM1_ESM.pdf]

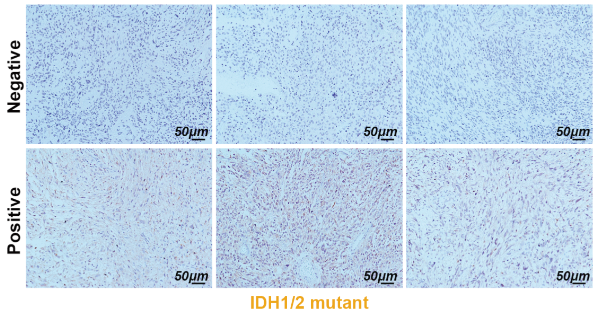

Fig. S1. Immunohistochemical staining of IDH1/2 mutant in tissue microarray of human brain tumour.

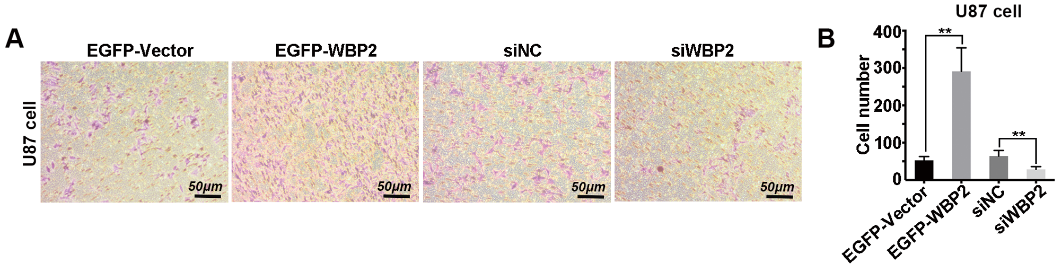

Fig. S2. Effects of WBP2 on the migration rate of GBM. (A) Efficiency of WBP2 on the migration ability in U87 cells was determined by transwell assay. Scale bar, 50 μm. **(B)** Cells on the lower surface of the membrane quantified and analyzed with five randomly selected fields. $**P<0.01$.

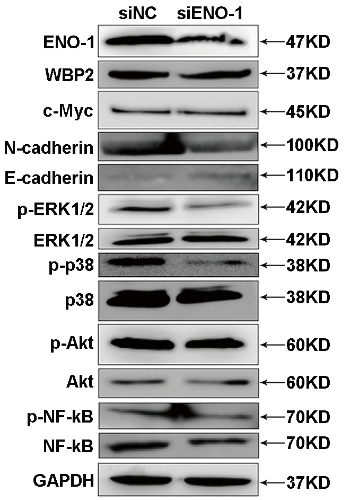

Fig. S3. Effects of ENO1 on the expression of WBP2 and the downstream signaling pathway.

Supplementary Table 1 Primers for quantitative real-time PCR

Gene	Forward sequence (5'-3')	Reverse sequence (5'-3')
**WBP2**	GCGGAGTGATCGTCAATAACA	GACCCGGTAAGGGGTAAGGT
**ENO1**	GCCTCCTGCTCAAAGTCAAC	AACGATGAGACACCATGACG
**Gapdh**	GGAGCGAGATCCCTCCAAAAT	GGCTGTTGTCATACTTCTCATGG
